# Revisiting the largest human leishmaniasis outbreak in Western Europe (Madrid, Spain): a follow-up entomological study in 2023 and 2024

**DOI:** 10.1186/s13071-026-07336-x

**Published:** 2026-04-11

**Authors:** José Risueño, Elena Verdú-Serrano, Matranga Alessandra, Pedro Pérez-Cutillas, Ricardo Molina, Maribel Jimenez, Inés Martín-Martín, Stefania Porcelli, Florence Robert-Gangneux, Jesús Veiga, Jordi Figuerola, Josué Martínez‐de la Puente, Eduardo Berriatua

**Affiliations:** 1 Facultad de Veterinaria, Departamento de Sanidad Animal, Campus de Espinardo, Regional Campus of International Excellence ‘Campus Mare Nostrum’, Universidad de Murcia, Espinardo, 30100 Murcia, Spain; 2 Dipartimento di Scienze Veterinarie, Polo Universitario dell’Annunziata, Universitá Degli Studi di Messina, 98168 Messina, ME Italia; 3 Facultad de Letras, Departamento de Geografía, Universidad de Murcia, Campus de la Merced, 30001 Murcia, Spain; 4 Laboratorio de Entomología Médica, Centro Nacional de Microbiología, and CIBERINFEC, Instituto de Salud Carlos III, Madrid, Spain; 5 Inserm, EHESP, Institut de Recherche en Santé, Environnement et Travail (Irset), UMR_S 1085, Univ Rennes, Rennes, France; 6 CHU Rennes, Inserm, EHESP, Irset, UMR_S 1085, Univ Rennes, Rennes, France; 7 Estación Biológica de Doñana, Consejo Superior de Investigaciones Científicas, (EBD‐CSIC), C\ Américo Vespucio 26, 41092 Seville, Spain; 8 Ciber de Epidemiología y Salud Pública (CIBERESP), Madrid, Spain

**Keywords:** Sand fly surveillance, Madrid, Leishmaniasis outbreak, *Phlebotomus*, *Leishmania infantum*

## Abstract

**Background:**

During the 2010s, Madrid experienced an outbreak of human leishmaniasis by *Leishmania infantum*, which affected at least 824 people until 2024 and peaked between 2010 and 2012. It was associated with a high density of infected *Phlebotomus perniciosus* sand fly vectors and, unusually, with rabbits and hares as the primary parasite reservoirs. In 2023 and 2024 we investigated sand fly species distribution, *L. infantum* infection rates, and bloodmeal sources in the outbreak area and compared the results with a similar study conducted in 2012–2014.

**Methods:**

The four sand fly surveillance sites used in 2012–2014 were revisited monthly between April and November 2023 and 2024. Sand flies were collected using CDC light traps and morphologically identified. *Leishmania* spp. infection and bloodmeal sources in female sand flies were molecularly tested.

**Results:**

In total, 5429 sand flies (2542 in 2023 and 2887 in 2024, 39% female) were collected, peaking in August and September. Overall, 72% of the captures were of *P. perniciosus*, 28% *Sergentomyia minuta*, < 1% *Phlebotomus ariasi*, and < 1% *Phlebotomus langeroni*. *Leishmania infantum* was confirmed in *P. perniciosus* and the estimated infection prevalence (95% CL) was 10% (7–13%). Blood sources of *P. perniciosus* were 80% (*n* = 51) rabbits, 11% (*n* = 7) humans, 6% (*n* = 4) hares, and 3% (*n* = 2) cats. Overall, 25%, 20%, and 19% of sand flies containing hare, human, and rabbit blood were *Leishmania* PCR-positive, respectively. The annual density of *P. perniciosus* in 2023–2024 was approximately half that observed in 2012 and comparable to levels in 2013 and 2014. In both 2012–2014 and 2023–2024 studies, *L. infantum* infection rates in *P. perniciosus* were similar, and lagomorphs accounted for most bloodmeals.

**Conclusions:**

Although the number of human leishmaniasis cases and the population of hares have been drastically reduced in the outbreak area, *P. perniciosus* densities and *L. infantum* infection rates in 2023–2024 in the sampling sites remain like those in 2012–2014; rabbits persist as a major bloodmeal source and probable reservoir of infection, maintaining human exposure to infected vectors. These results underscore the importance of sustained sand fly surveillance in this hotspot to inform public health decision-making and guide targeted control strategies.

**Graphical Abstract:**

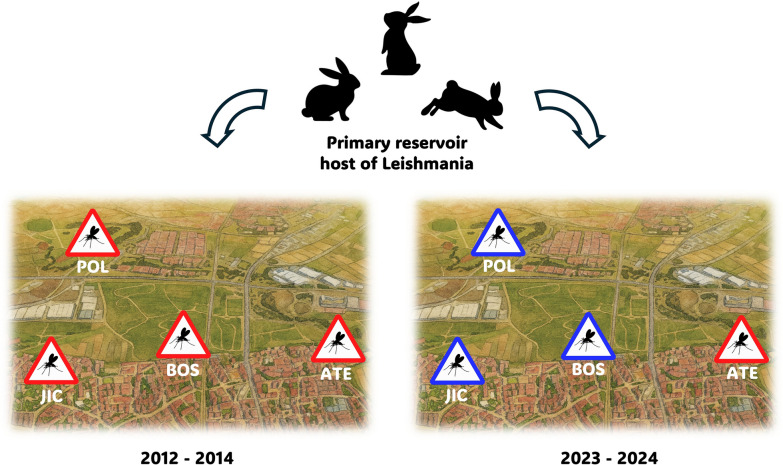

**Supplementary Information:**

The online version contains supplementary material available at 10.1186/s13071-026-07336-x.

## Background

Phlebotomine sand flies (Diptera: Psychodidae) are hematophagous insect vectors of *Leishmania* spp., a group of protozoan parasites affecting both animals and humans [2]. *Leishmania infantum* is the main pathogenic species in Europe, and domestic dogs are the primary reservoir hosts for this species. Dogs are highly susceptible to infection, often developing canine leishmaniasis (CanL), which is a deadly multisystemic chronic disease. Human leishmaniasis (HumL) is less common than CanL, and typical clinical presentations include visceral leishmaniasis (VL), a life-threatening condition, and cutaneous leishmaniasis (CL), which is generally a benign form. Typically, VL is associated with specific risk groups, such as young, malnourished children and immunocompromised adults, including those infected with human immunodeficiency virus (HIV) and organ transplant recipients [2].

Spain is considered a HumL hypoendemic country with a reported mean annual incidence in the period 2005–2020 below 1 case per 100,000 population [4]. Until 2009, the incidence of HumL in the Autonomous Community of Madrid (ACM) aligned with the national average, with only nine cases reported that year in the southwestern region (SW Madrid), the focus area of the present study [1]. However, between 2010 and 2016, the number of HumL cases in SW Madrid increased to 691, with the highest incidence reported in the town of Fuenlabrada (45 cases per 100,000 population) [5]. No outbreak of HumL of comparable magnitude had previously been recorded in Europe. Unusually, most affected individuals were immunocompetent adults, with 36% developing VL and 64% presenting with CL lesions [1]. Following the implementation of an intense environmental control program targeting hosts and vectors, incidence decreased and the number of HumL cases reported in ACM between 2019 and 2021 was 113 cases [6]. Between 2021 and 2024, 48 HumL cases were reported in SW Madrid (Dirección General de Salud Pública, Madrid, personal communication). Notably, the HumL outbreak was not accompanied by a corresponding rise in the incidence of CanL [7]. Instead, it was linked to an overpopulation of hares and rabbits in the periurban Bosquesur recreational park, which acted as an unusual reservoir for *L. infantum*, along with a high density of *L. infantum*-infected *Phlebotomus perniciosus*, which fed primarily on lagomorphs [8–12].

Here, we compare key entomological and epidemiological information on HumL in SW Madrid collected in 2023 and 2024 with those recorded between 2012 and 2014 in a similar entomological study [5]. The objective is to test the hypothesis that the substantial decline in HumL incidence in this area in recent years is associated with a reduction in sand fly density and *L. infantum* infection rates.

## Methods

### Study design and area

This study was part of a broader entomological and epidemiological survey of sand flies across Spain and other European regions within the EU-funded CLIMOS project (https://climos-project.eu/). The sand fly sampling sites selected in Madrid were the same as those used in a previous survey conducted by González et al. [5] to allow comparability. These locations were originally selected on the basis of high sand fly densities observed during a preliminary survey in 2011.

**Fig. 1:**
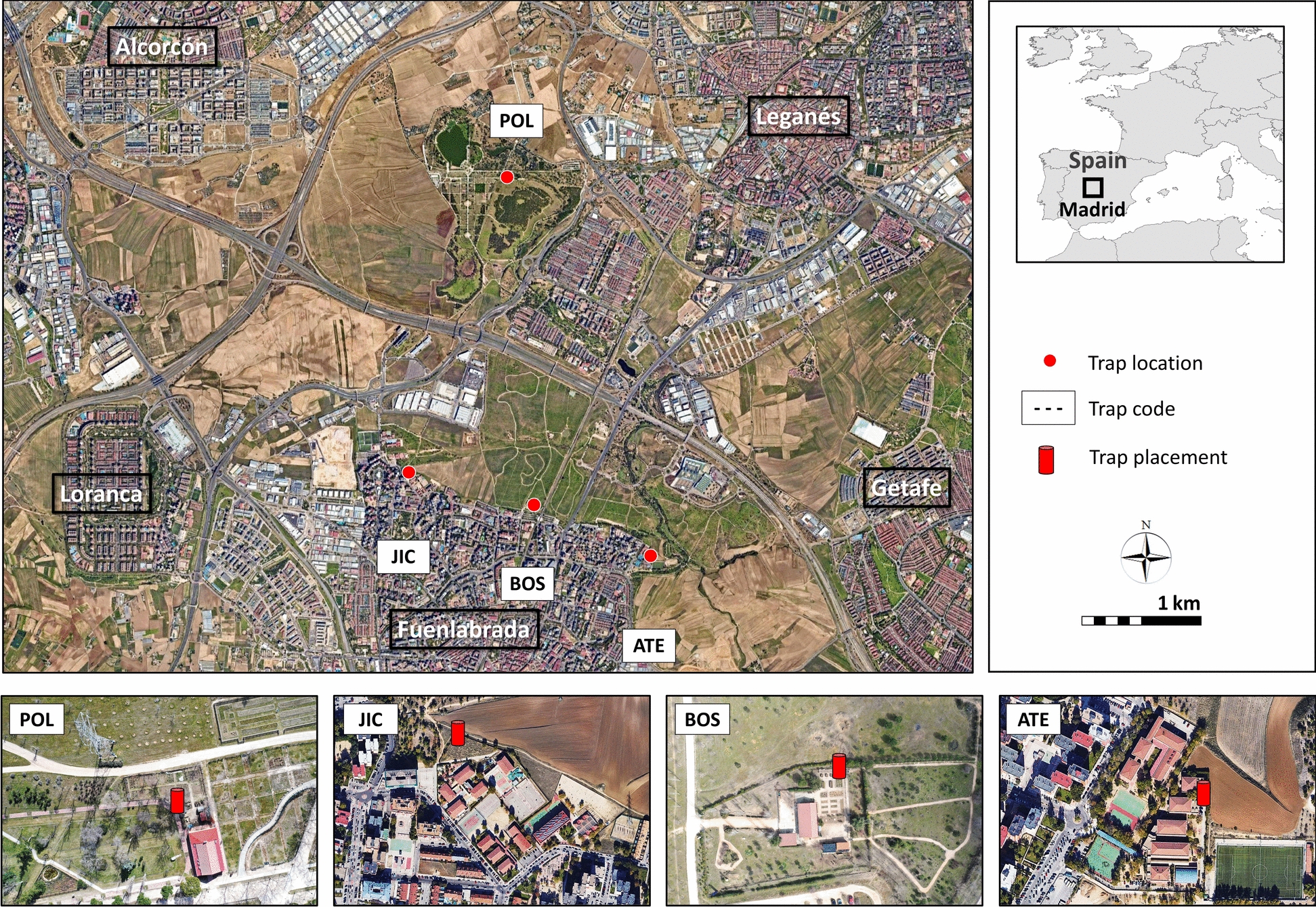
Entomological study and trap placement sites in southwest Madrid

Sand fly surveillance was conducted from April to November in 2023 and 2024 at four fixed locations: three in Fuenlabrada: ATE (−3.792931, 40.298234), BOS (−3.780526, 40.292838), and JIC (−3.806122, 40.300579), and one in Leganés: POL (−3,796,362 40324845) (Fig. 1; Supplementary Fig. 1S) in the southwestern (SW) region of the Madrid Metropolitan Area. Fuenlabrada (39 km^2^; 664 m above sea level (a.s.l.); population ~194,000) and Leganés (43 km^2^; 665 m a.s.l.; population ~191,000) reported the highest number of cases during the 2010s HumL outbreak in Madrid [1, 5]. The sampling locations included the outdoor grounds of two secondary schools (ATE and BOS) and two environmental education centers (JIC and POL). Sites ATE, BOS, and JIC are situated at the edge of urban residential areas adjacent to Bosquesur recreational park. In contrast, POL is located inside this park (Fig. 1; Supplementary Fig. 1S).

Land cover within 50 m and 500 m buffers around traps was assessed using 2023 PNOA aerial imagery in QGIS v3.34 (Supplementary Fig. 2S). At 50 m, ATE was most urbanized, BOS dominated by pasture and bush, POL by forest, and JIC showed mixed covers of cropland, pasture, and forest. At 500 m, site differences diminished (Supplementary Fig. 2S).

### Sand fly surveillance and morphological identification

The sampling strategy closely followed that of González et al. [5], with both studies employing miniature Centers for Disease Control (CDC) battery-operated light-aspiration traps (J.W. Hock Company, Gainesville, FL, USA) deployed for two consecutive days per month. However, some methodological differences existed. In the present study, 1 light trap per site was used monthly from April to November, whereas González et al. [5] used 2 light traps and 20 sticky traps per site, sampling from June to October.

Traps were set on the first day between 17:00 and 19:00, and collection cages were retrieved the following morning between 08:00 and 10:00. Fresh cages were then installed and collected again the next morning. Collected cages containing insects were transported to the laboratory in plastic bags with a ball of filter paper soaked in water. Upon arrival, the contents were sorted by trap and collection date into individual cages. Specimens were then separated on the basis of viability: dead individuals were preserved in 70% ethanol at −20 °C, while live specimens were stored dry at −80 °C.

Morphological identification of male and female specimens involved dissecting the head and the last two abdominal segments on cold plates and examining key anatomical features: the external genitalia in males, and the pharynx, cibarium, and spermatheca in females [3, 13]. The dissected parts were cleared using Marc André solution, mounted on glass slides with Hoyer’s medium, and examined at 400× magnification. The remaining body parts of the sand flies not used for morphological identification, thorax and abdomen, were preserved either in 70% ethanol (for specimens collected dead) or at −80 °C (for those collected alive) and later used for molecular analysis.

### Host species bloodmeal identification in sand flies

DNA from 114 blood engorged *P. perniciosus* female sand flies collected in 2023 (*n* = 24) and 2024 (*n* = 90) from ATE (*n* = 96), BOS (*n* = 9), and POL (*n* = 9) were analyzed to identify the vertebrate host species. A 648-base pair fragment of the cytochrome c oxidase subunit I (*COI*) gene was amplified and sequenced [14]. In brief, a nested PCR approach was employed: the initial amplification used M13BCVFW and BCV-RV1 primers, followed by a second run using M13BCV-FW and BCV-RV2 primers. The resulting PCR products were separated via electrophoresis on a 1.5% agarose gel, stained with SYBR Safe DNA Gel Stain (Invitrogen) and visualized under ultraviolet (UV) light. Amplicons were sequenced using the facilities of STAB VIDA (Caparica, Portugal) using bidirectional primers (M13BCV-FW and BCV-RV2). Sequence data were processed using Geneious software (Geneious Prime^®^ 2024.0.7) and the resulting sequences were queried against the GenBank (National Center for Biotechnology Information) database and the Barcode of Life Data Systems (BOLD systems) using the Basic Local Alignment Tool (BLAST). Vertebrate host identity was assigned when sequence similarity to known species was ≥ 98%.

### Detection of *Leishmania* spp. in sand flies

*Leishmania* spp. infection was investigated in 402 individual female sand flies including 394 *P. perniciosus*, 5 *Sergentomyia minuta*, and 2 *Phlebotomus ariasi*, from ATE (*n* = 162), BOS (*n* = 83), JIC (*n* = 81), and POL (*n* = 76). Selected samples included most *P. perniciosus* females containing blood, eggs, or both, and a random selection of females with empty abdomens (non-gravid and non-blood-fed) from the four sites, allowing for approximately twice as many sand flies from ATE (where 71% of sand flies were collected) than from the other sites, to obtain a more precise estimation of *Leishmania* infection rates at this site. We used a TaqMan real-time polymerase chain reaction (qPCR) assay targeting an ~120 base pair (bp) region of the *Leishmania* kinetoplast minicircle DNA (*kDNA*) [15] and reactions were conducted in duplicate for 40 amplification cycles. DNA samples used were from the thorax and abdomen of individual females, homogenized in 500 µL of phosphate-buffered saline (PBS) using disposable plastic pestles. DNA was extracted from dead specimens using the 16 LEV Blood DNA Kit (Promega, ref. AS1290) and from live specimens using the 16 Viral Total Nucleic Acid Purification Kit (Promega, ref. AS1150), in the Maxwell semi‐automated nucleic acid purification robot (Promega). DNA from colony-derived *L. infantum*-infected and uninfected sand flies from Charles University, Prague, were used regularly as positive and negative controls, respectively.

The extracted DNA was quantified using a spectrophotometer (Implen NanoPhotometer). Parasite loads in PCR-positive sand flies were semi-quantitatively estimated by determining the mean threshold cycle (CT) from the PCR amplification curves of each sample. The CT value was identified as the point where the first signs of near-logarithmic amplification appeared. Samples with CT values < 30 were considered strong positives, those with CT values between 33 and 38 were classified as low-level positives, and samples with CT values ≥ 38 were considered very low-level positives, as these values are close to the assay’s limit of detection and likely reflect very low target quantities for which quantitative interpretation is unreliable [16].

### Species-level identification of *Leishmania* spp.

*Leishmania* species were identified by *Hsp70* gene amplification and sequencing in DNA from 35 of 43 PCR-positive sand flies with sufficient DNA, including 34 *P. perniciosus* and 1 *S. minuta*, following the protocol described by Van Der Auwera et al. (2013) [27] and using GoTaq^®^ G2 Hot Start Polymerase (Promega, France). For samples failing initial sequencing attempts, a second PCR targeting a distinct *Hsp70* fragment (PCR-C) was conducted, utilizing the first PCR product (PCR-F) as a template [27]. PCR amplicons were subsequently purified and sequenced with internal primers employing the BigDye Terminator v3.1 kit (Applied Biosystems, France) on an ABI Prism 3130XL sequencer. Multiple sequence alignments were then carried out using the SeqScape software. The aligned consensus sequences were visually inspected and edited as needed for accuracy. For species identification and confirmation, sequences were compared against reference sequences using the BLAST tool from the NCBI database, and aligned with reference sequences in MEGA5 software, using the bootstrap method [17]. The sets of primers used in these analyses are described in Supplementary Table 1S.

### Statistical analysis

The distribution of study variables was investigated including the proportion of traps with sand flies (positive traps), sand fly abundance (number of sand flies), density (number of sand flies per trap and night), and sand fly *Leishmania* infection prevalence (% of *Leishmania* PCR positives).

Bivariate relationships were then analyzed using the Chi-squared test, or when appropriate, Fisher’s exact test, to compare proportions, and the Kruskal–Wallis nonparametric test to compare medians. Statistical significance was assessed at *α* = 0.05 using two-tailed tests [18]. Analyses were carried out in R statistical software [22].

## Results

### Sand fly distribution and species diversity and seasonality

A total of 5429 sand flies were collected in 85 of 144 trap-nights between April and November 2023–2024, with no captures in April or November (Table 1). Overall, 39% were female and 61% male, with a significantly higher proportion of females in 2023 (42%) than in 2024 (36%). Abundance varied among sites, with ATE contributing the majority of captures (1849 in 2023 and 2031 in 2024), followed by BOS (236 and 368), JIC (185 and 281), and POL (272 and 207) (Table 1).

**Table 1:** Proportion of traps with sand flies and sand fly distribution in positive traps according to explanatory variables

Variable	Level	Traps			Sand flies								
		Total	Positive (%)	*P*-value	Total	Density*	Mean	Minimum	Q1	Median	Q3	Maximum	*P*-value
Sex	Female	144	59	0.4057	2121	15	27	1	3	10	29	222	0.3098
	Male	144	53		3298	23	43	1	5	15	33	440	
Year	2023	72	61	0.7347	2542	35	58	1	5	23	52	520	0.6252
	2024	72	57		2887	40	70	1	11	22	66	515	
Site	ATE	36	69	< 0.0001	3880	108	155	1	23	75	188	520	0.0010
	BOS	36	61		604	17	27	1	6	16	26	159	
	JIC	36	50		466	13	26	1	3	15	48	93	
	POL	36	56		479	13	24	1	7	18	35	102	
Month	April	16	0	< 0.0001	0	0	0	0	0	0	0	0	0.0004
	May	16	44		61	4	9	1	1	1	12	33	
	June	16	75		350	22	29	1	4	14	36	149	
	July	24	96		1232	51	54	1	14	23	58	299	
	August	24	100		2888	120	120	2	26	50	113	520	
	September	16	94		868	54	58	1	3	11	43	474	
	October	16	25		30	2	8	3	4	5	8	18	
	November	16	0		0	0	0	0	0	0	0	0	
All		144	59		5429	38	64	1	5	22	61	520	

^*^Density: number of specimens per trap, per night

Most specimens (98%) were identified to species level. Species diversity was dominated by *P. perniciosus* (72%), followed by *S. minuta* (28%), with *P. ariasi* and *P. langeroni* each accounting for < 1%. Species relative abundances were similar between years but varied among sites, with *P. perniciosus* comprising 79%, 75%, 61%, and 55% of captures in BOS, ATE, POL, and JIC, respectively.

**Fig. 2:**
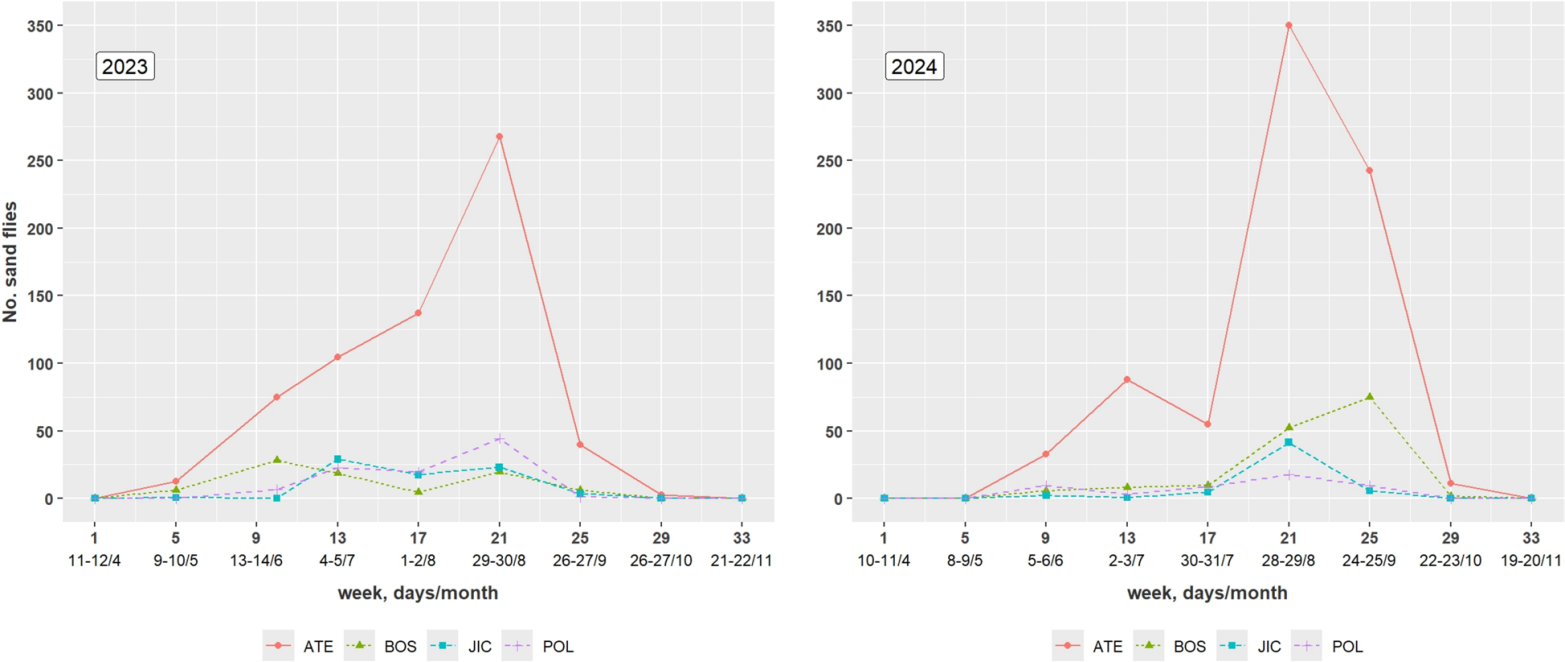
Seasonal distribution of *Phlebotomus perniciosus* sand fly density (No. specimens per trap, per night) in 2023 and 2024 in ATE, BOS, JIC, and POL study sites in southwest Madrid

Sand fly densities were strongly seasonal, increasing in mid-June/early July and peaking in August, with high densities persisting into September 2024. Seasonal trends were driven primarily by *P. perniciosus* (Fig. 2), while *S. minuta* followed a similar but lower pattern, and few *S. minuta* specimens were collected in September 2024.

### Comparison of present and past annual density of sand flies

To allow comparison between study periods, sand fly abundance was expressed as density (number of sand flies per trap-night) using CDC light-trap data from May to October only.

Overall, *P. perniciosus* densities in 2023–2024 were lower than those reported in 2012 but comparable to 2013–2014, with marked spatial heterogeneity (Table 2). Densities at ATE remained high and similar to 2012, whereas BOS, JIC, and POL showed substantially reduced densities relative to 2012–2014. In contrast, *S. minuta* densities were higher in 2023–2024 than in the earlier study period (Table 2). Seasonal activity patterns were consistent across both studies, with densities increasing from June and peaking in late summer, despite interannual variation (Fig. 2) [5].

**Table 2:** Annual density of *Phlebotomus perniciosus* and *Sergentomyia minuta* in ATE, BOS, JIC, and POL study sites in the present study (2023 and 2024) and in González et al. [5] (2012, 2013, and 2014)*

Year	*Phlebotomus perniciosus*					*Sergentomyia minuta*				
	ATE	BOS	JIC	POL	All	ATE	BOS	JIC	POL	All
2012	295	79	321	110	201	23	1	9	11	11
2013	92	98	102	55	87	1	2	7	1	3
2014	97	163	115	29	101	9	2	6	3	5
2023	251	31	29	38	87	99	11	7	16	33
2024	312	61	22	19	103	92	12	35	20	40

^*^Data from 2012 to 2014 was obtained from the summary statistics of light trap collections only, provided in Fig. 2 [5]. To account for sampling differences between studies, comparisons are for CDC light trap collections between May to October and sand fly densities are expressed as the number of specimens per trap per night

### Identification of vertebrate hosts of sand flies

Identification of the vertebrate host species of the 114-blood engorged *P. perniciosus* analyzed was successful in 64 specimens (11 from 2023 and 53 from 2024), including 57 from ATE, 3 from BOS, and 4 from POL.

Blood sources corresponded to 51 (80%) rabbits (*Oryctolagus cuniculus*), 7 (11%) humans (*Homo sapiens*), 4 (6%) hares (*Lepus* spp.), and 2 (3%) cats (*Felis catus*/*Felis silvestris*). Rabbit blood was detected at all three sites in both years. Human blood was identified at ATE and BOS in both years, hare blood at ATE and POL in 2024, and cat blood at POL in both years.

### *Leishmania* spp. infection rates and identification in sand flies

*Leishmania* spp. DNA was detected in 43 of 402 tested sand flies, corresponding to an overall PCR prevalence of 11% (95% CI 8–14%). Prevalence was significantly higher in 2024 than in 2023 (16% versus 4%) and varied among sites, with the highest values observed at ATE (16%) (Table 3).

**Table 3:** Percentage (95% confidence limits) of PCR-positive sand flies and number of positives according to PCR threshold cycle (CT) ranges by levels of explanatory variables

Variable	Level	Sand flies					PCR CT ranges			
		No	Positives (%)	95 CL−	95 CL+	*P*-value	16–28	33–38	39–40	*P*-value
Site	ATE	162	16	10	22	0.0315	6	17	3	0.5723
	BOS	83	5	0	9		0	3	1	
	JIC	81	9	3	15		0	5	2	
	POL	76	8	2	14		0	5	1	
Abdominal	Blood	88	14	6	21	< 0.0001	3	7	2	0.1623
Contents	Blood and eggs	11	36	8	65		2	2	0	
	Eggs	37	27	13	41		1	7	2	
	Empty	266	6	3	9		0	14	3	
Sand fly species	*P. perniciosus*	395	10	7	13	0.1661	6	29	6	0.5183
	*P. ariasi*	2	0	0	0		0	0	0	
	*S. minuta*	5	40	0	83		0	1	1	
Host species blood	*Oryctolagus cuniculus*	47	19	8	30	1.0000	4	4	1	1.0000
	*Lepus* spp.	4	25	0	67		0	1	0	
	*Felis catus*/*F. silvestris*	2	0	0	0		0	0	0	
	*Homo sapiens*	5	20	0	55		0	1	0	
Year	2023	179	4	1	7	0.0002	0	6	1	0.8143
	2024	223	16	11	21		6	24	6	
Month	May	5	0	0	0	0.0926	0	0	0	0.6614
	June	23	9	0	20		0	1	1	
	July	78	3	0	6		0	2	0	
	August	203	13	9	18		5	19	3	
	September	90	13	6	20		1	8	3	
	October	3	0	0	0		0	0	0	
All		402	11	8	14		6	30	7	

A weak seasonal pattern was observed, with higher prevalence in early and late summer (Table 3). Infection was strongly associated with female physiological status, with higher prevalence in females with eggs (with or without blood) compared with blood-fed-only or empty females. Infections were detected in sand flies containing rabbit (19%), hare (25%), and human blood (20%) (Table 3).

Threshold cycles in PCR-positive sand flies ranged between 16 and 40, and most PCR-positive specimens had high CT values (≥ 33) (Table 3), consistent with low parasite quantities. The lowest range CT values, indicating large parasite loads, were found in blood-fed and gravid *P. perniciosus* females from ATE in August and September 2024, and were associated with rabbit bloodmeals.

Successful *Hsp70* amplification and sequencing was limited to five *P. perniciosus* specimens with low CT values (16–28), all of which were identified as *Leishmania infantum*.

## Discussion

The results from this study evidence an ongoing *L. infantum* transmission cycle in SW Madrid, mostly in the outdoor grounds of a secondary school in Fuenlabrada (ATE area), where a large density of infected *P. perniciosus* vectors was detected, and highlight the need for wider sand fly surveillance in this area. Overall, our study findings have significant similarities with those reported by González et al. [5] in their study conducted in 2012–2014. However, comparisons between studies should consider methodological differences, as this CLIMOS-based study sampled between April and November instead of May to October in 2012–2014, and owing to logistical constraints, relied exclusively on CDC light traps and a single outdoor trap per site. To address differences in sampling effort between studies, sand fly abundance was calculated for the same periods and expressed as density per trap-night. Species richness was nearly identical, with a clear predominance of *P. perniciosus*, whose densities peaked in August and September. Infection rates were also similar (13% in 2012–2014 and 11% in 2023–2024), with the highest values occurring in August and September. In both studies there was substantial variation between years in these parameters, and in the sex ratios in 2023–2024, and this warrants further investigation. The most remarkable difference was the comparatively lower density and infection rates in sand flies in BOS, JIC, and POL in 2023–2024 compared with 2012–2014, and to a previous study in JIC in 2011 [19]. In addition, hares were the main blood source for sand flies in 2012–2017 [20], whereas rabbits predominated in 2023–2024. This suggests that control interventions by public health authorities in these areas have been effective. Early interventions during the winter of 2010–2011 included waste removal, elimination of accumulated organic matter as potential sand fly breeding and resting sites, and the application of insecticides in vector hotspots [29]. Once hares were confirmed as reservoirs of *L. infantum* involved in the outbreak [8], their populations in the park area surrounding the BOS and POL sites were drastically reduced in early 2012. The implementation of control activities led to a 56% decline in leishmaniasis incidence from July 2012 to June 2013. Similar measures were later applied to control rabbit populations in these areas after this species was also identified as a reservoir in 2013 [9]. No control of the lagomorph population was carried out in the immediate area around ATE. This could explain the large abundance of sand flies with rabbit and human bloodmeals and the high proportion of PCR positives, most with low and moderate CT values, indicating large parasite loads, at this locality. Further investigation is needed to assess the risk of leishmaniasis for residents of the ATE area, beginning with a preliminary analysis linking current cases to their place of residence.

Insecticides were not applied at trapping sites during the present study, and the high density of sand flies in ATE compared with other sites are most probably associated with specific environmental features favorable for sand flies. Sand flies typically have small dispersal areas, concentrating their activity in the proximity of bloodmeal sources [21]. The ATE sampling locality was distinctive in terms of land cover within the 50 m radii buffer, with 40% urban cover (versus 0–2% at other sites) and the remaining 60% of the surface classified as cropland (compared with 37% at JIC and 0% at BOS and POL). Rabbits were present in the cropland, while the urban portion—being part of a secondary school—was frequented by people. Furthermore, the ATE trap site was sheltered and surrounded by dense vegetation, likely offering optimal conditions for sand fly development [23] (Supplementary Fig. 2S).

Sand flies are opportunistic blood feeders and may feed on a wide range of vertebrate host species [20, 24]. Bloodmeal sources and *Leishmania* spp. burdens in infected sand flies have key epidemiological implications because host species differ in their capacity to maintain and transmit the parasite to the vector. In xenodiagnostic experiments, rabbits and hares were efficient transmitters of *L. infantum* [8, 9], whereas immunocompetent humans did not transmit the infection to sand flies, in contrast to infected immunosuppressed individuals, who did [25]. The role of cats in the *L. infantum* transmission cycle is less clear [26], and in similar studies, transmission to vectors occurred only in sand flies feeding on cats with symptoms or biochemical alterations compatible with feline leishmaniosis (FeL) [28]. Screening of a large cohort of stray cats in Madrid during the HumL outbreak period revealed minimal evidence of FeL [30]. Also, the low population density of cats in Bosquesur park suggests they are unlikely to play a significant epidemiological role in this area. However, it is necessary to consider that the infection’s origin in blood-fed females may not always correlate with the host species of the current blood meal. This is because sand flies may feed several times and on several hosts, and digest the blood and produce and lay eggs between meals, while maintaining infection from a first feed. Nonetheless, all low CT counts (indicative of high parasite burdens) were detected in sand flies with rabbit blood, supporting rabbits —together with hares— as primary reservoirs of *L. infantum* in SW Madrid [12]. Vectors carrying high *Leishmania* burdens exhibit greater transmission efficiency and a higher likelihood of triggering epidemics in humans than those with low infective doses [31].

Sequencing PCR-positive samples was successful only for those with low CT values, confirming the presence of *L. infantum* in these cases. The limited success in sequencing high CT positives is likely because of the relatively low sensitivity of the *Hsp70*-PCR compared with the *kDNA*-PCR used for diagnosis. *Hsp70* sequences are widely used in phylogenetic and taxonomic studies of *Leishmania* owing to inter-species polymorphisms, whereas kinetoplast minicircle sequences are well suited for detecting infections with minimal parasite loads owing to their high copy number [32]. Mary et al. [15] reported a kPCR detection limit of 0.0005 parasites per reaction tube, determined using serial dilutions of parasite DNA from a known parasite count. Future efforts will therefore focus on samples with high CT values, targeting alternative, more sensitive genetic markers to improve species identification in low-parasite-load samples. *Leishmania infantum* was the only *Leishmania* species identified in the leishmaniasis outbreak in Madrid [5, 12] and, with rare exceptions, it is the only endemic pathogenic species in Europe [2]. However, recent reports have documented the presence of other *Leishmania* spp. in Spain, including *L. tropica*— an endemic species in Northern Africa, the Middle East, and Turkey—identified in two wild cats [33], as well as *Leishmania tarentolae* and *Leishmania adleri*—two Sauroleishmania species of reptiles — detected in their natural vector *S. minuta* [34, 35]. Notably, *L. tarentolae* DNA was also detected in blood samples from humans, dogs, and cats in Italy, suggesting that mammals could also become infected [36, 37]. In addition, *S. minuta* has been shown to readily feed on humans and other mammal species [38].

## Conclusions

Although the number of HumL cases has been drastically reduced in the outbreak area, the ecological and epidemiological conditions that support transmission persist. Consequently, the results of this study emphasize the need of sustained, active, and long-term surveillance as an essential component of leishmaniasis control in southwest Madrid, Spain. Continued active entomological and epidemiological monitoring is needed to detect changes in sand fly populations and in infection dynamics to prevent disease re-emergence.

Particular attention should be given to the area surrounding the ATE sampling station, where entomological studies should be integrated with epidemiological investigations to better link reported human cases with residential locations. Building on previous findings, an effective leishmaniasis control strategy should include reducing the density of lagomorph reservoirs before the onset of sand fly season in May. This measure may be more challenging in ATE, where rabbits concentrate in cropland, compared with BOS and POL, where rabbits and hares were more accessible in the recreational park. Finally, the information generated in this study will also support broader research aimed at improving understanding of the impacts of climate change on sand fly populations and sand fly-borne infections, further supporting the need for sustained surveillance and adaptive, targeted public health interventions.

## Supplementary Information

Supplementary Material 1.

## Data Availability

Data generated in this study are available within the manuscript or as additional supplementary files to this manuscript.
