# Supplementary material for: Revisiting the largest human leishmaniasis outbreak in Western Europe (Madrid, Spain): a follow-up entomological study in 2023 and 2024
Source: Parasit Vectors. 2026 Apr 11;19:222. doi: 10.1186/s13071-026-07336-x (PMC13185400; doi:10.1186/s13071-026-07336-x)
Supplement: Supplementary file 1 — Supplementary Material 1. [file 13071_2026_7336_MOESM1_ESM.docx]

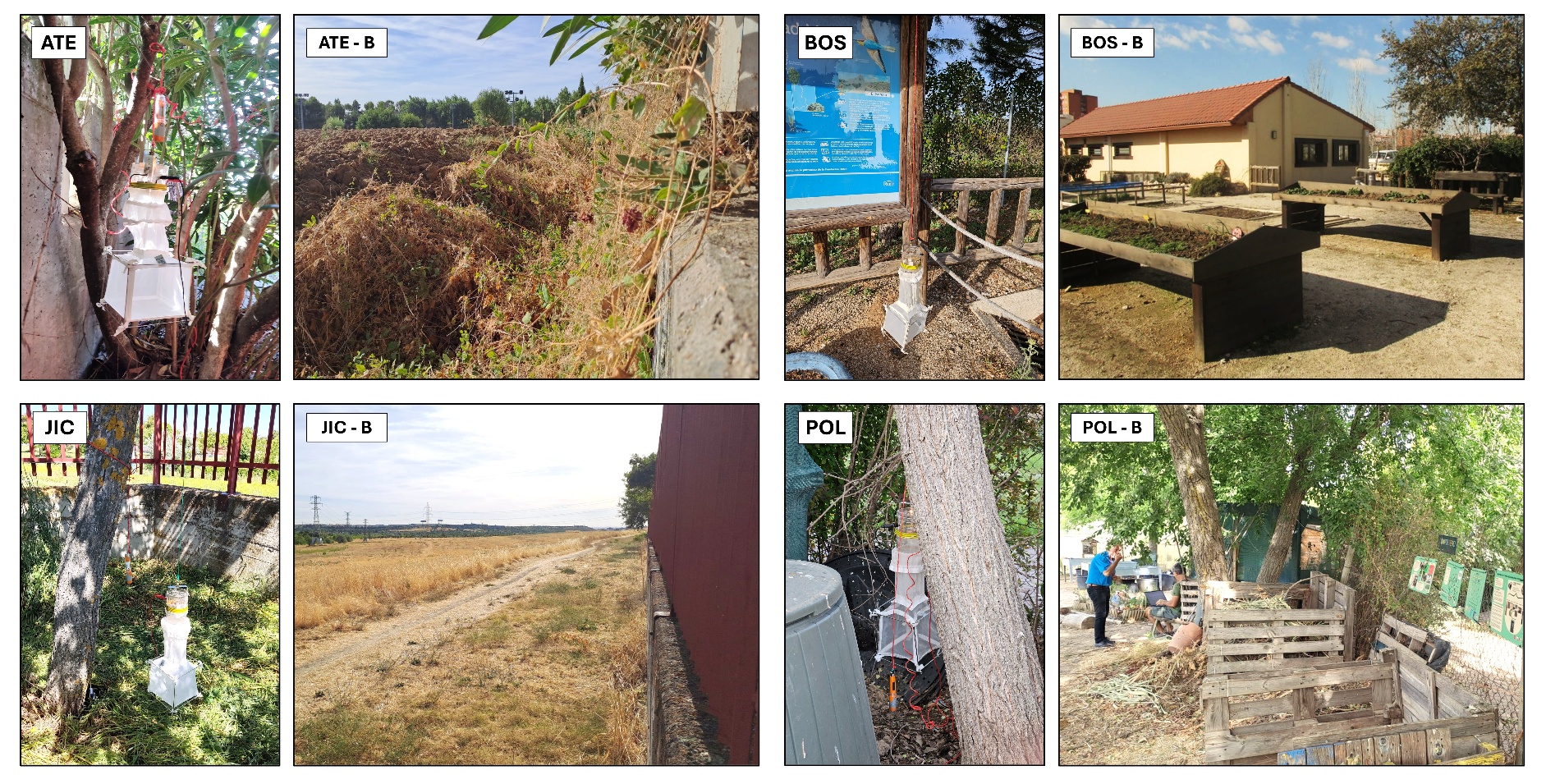


Fig. 1S. Representative photographs of ATE, BOS, JIC and POL sampling sites included in this study. For each site, two images are provided: the image on the left corresponds to the exact trap placement and the image on the right illustrates the surrounding environment. In the case of ATE-B, the photo was taken from outside the perimeter fence where the trap was installed, depicting a dense patch of spontaneous vegetation acting as a boundary to a cultivated area. BOS-B shows the front side of the trap, located within a garden adjacent to the main building of the Bosquesur Park Environmental Education Centre. JIC-B was taken from the outer side of the fence enclosing the trap, revealing a vegetation-free dirt path with farmland visible in the background. Lastly, POL-B captures the plant storage and maintenance area of the Polvoranca Environmental Education Centre, located within the Bosquesur Park, where the trap was integrated among the surrounding materials and vegetation.


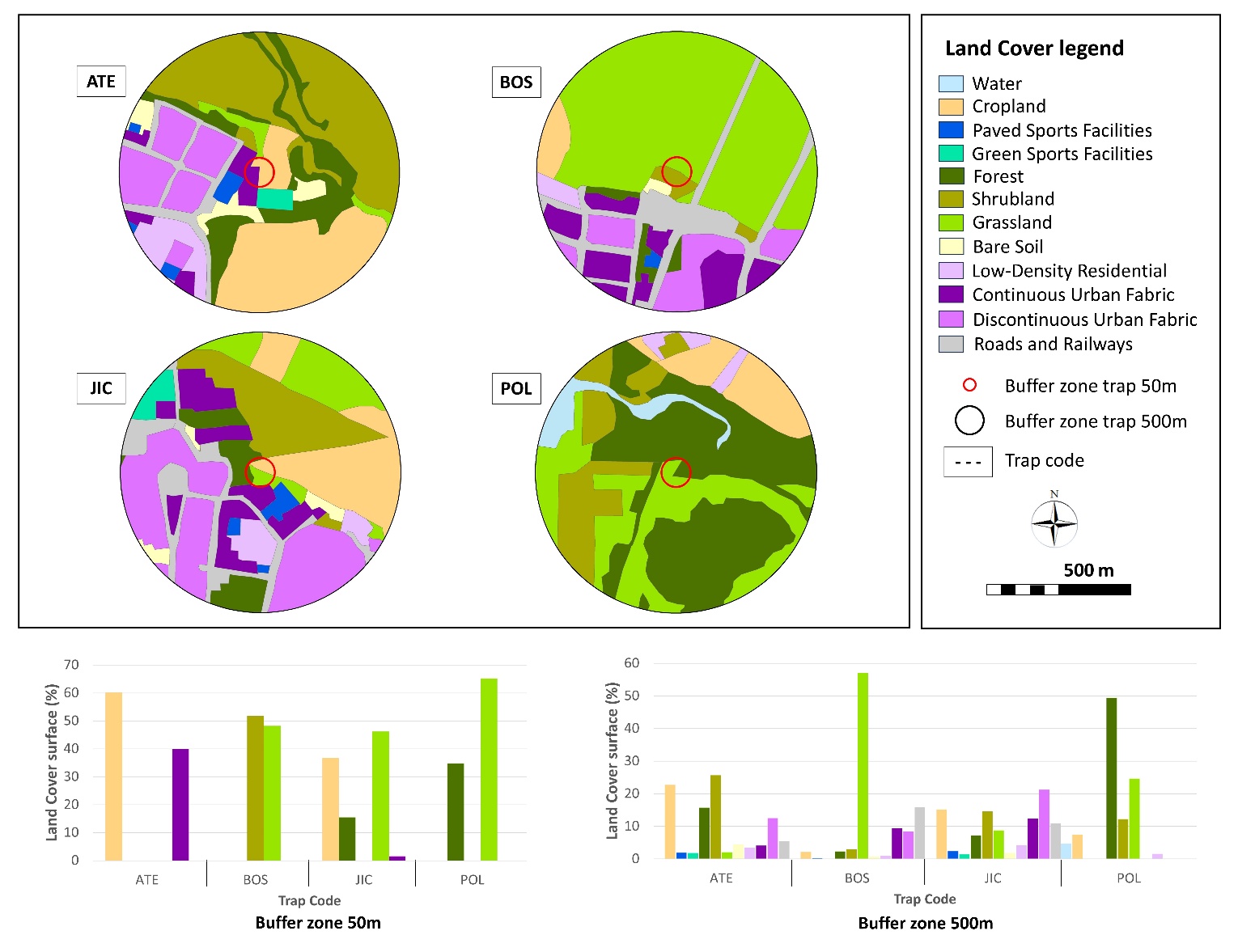


Fig. 2S. Predominant land cover types in circular buffer areas with radii of 50 m and 500 m around each trap, estimated through photointerpretation of aerial imagery from the 2023 National Plan for Aerial Orthophotography (PNOA).

Table 1S. PCR primers used for the amplification of a partial coding region of the *hsp70* gene of *Leishmania* spp.

| Primer name /PCR | Sequence (5’-3’) |
| --- | --- |
| HSP70-F25 (PCR-F/N) | 5’-GGACGCCGGCACGATTKCT |
| HSP70-R617 (PCR-N) | 5’-CGAAGAAGTCCGATACGAGGGA |
| HSP70-R1310 (PCR-F) | 5’-CCTGGTTGTTGTTCAGCCACTC |
| HSP70-4F (PCR-F, N) | 5’- CACGATTKCTGGSCTGGAGGTG |
| HSP70-4R (PCR-N) | 5’-GACTGCACCTTCGGGATGCG |
| HSP70-6R (PCR-F) | 5’-GTTGTTGTTCAGCCACTCCAGC |
| HSP70-F251 (PCR-C) | 5’- GACAACCGCCTCGTCACGTTC |
| HSP50-R991 (PCR-C) | 5’- GTCGAACGTCACCTCGATCTGC |
